# Multi-responses of O-methyltransferase genes to salt stress and fiber development of Gossypium species

**DOI:** 10.1186/s12870-020-02786-6

**Published:** 2021-01-11

**Authors:** Abdul Hafeez, Qún Gě, Qí Zhāng, Jùnwén Lǐ, Jǔwǔ Gōng, Ruìxián Liú, Yùzhēn Shí, Hǎihóng Shāng, Àiyīng Liú, Muhammad S. Iqbal, Xiǎoyīng Dèng, Abdul Razzaq, Muharam Ali, Yǒulù Yuán, Wànkuí Gǒng

**Affiliations:** 1 State Key Laboratory of Cotton Biology, Institute of Cotton Research, Chinese Academy of Agricultural Sciences, Anyang, 455000 Henan China; 2 Sindh Agriculture University Tandojam, Hyderabad, Sindh 70060 Pakistan

**Keywords:** O-methyltransferase, *Gossypium*, Fiber development, Biotic and abiotic stress

## Abstract

**Background:**

O-methyltransferases (OMTs) are an important group of enzymes that catalyze the transfer of a methyl group from S-adenosyl-L-methionine to their acceptor substrates. OMTs are divided into several groups according to their structural features. In *Gossypium* species, they are involved in phenolics and flavonoid pathways. Phenolics defend the cellulose fiber from dreadful external conditions of biotic and abiotic stresses, promoting strength and growth of plant cell wall.

**Results:**

An *OMT* gene family, containing a total of 192 members, has been identified and characterized in three main *Gossypium* species, *G. hirsutum*, *G. arboreum* and *G*. *raimondii*. Cis-regulatory elements analysis suggested important roles of *OMT* genes in growth, development, and defense against stresses. Transcriptome data of different fiber developmental stages in Chromosome Substitution Segment Lines (CSSLs), Recombination Inbred Lines (RILs) with excellent fiber quality, and standard genetic cotton cultivar TM-1 demonstrate that up-regulation of *OMT* genes at different fiber developmental stages, and abiotic stress treatments have some significant correlations with fiber quality formation, and with salt stress response. Quantitative RT-PCR results revealed that *GhOMT10_Dt* and *GhOMT70_At* genes had a specific expression in response to salt stress while *GhOMT49_At*, *GhOMT49_Dt*, and *GhOMT48_At* in fiber elongation and secondary cell wall stages.

**Conclusions:**

Our results indicate that O-methyltransferase genes have multi-responses to salt stress and fiber development in *Gossypium* species and that they may contribute to salt tolerance or fiber quality formation in *Gossypium*.

**Supplementary Information:**

The online version contains supplementary material available at 10.1186/s12870-020-02786-6.

## Background

Cotton (*Gossypium* Species) has the importance for natural fiber all over the globe. The primary goals of upland cotton (*G. hirsutum*) perspectives have been always to achieve better quality with higher yield [1]. Mostly *G. hirsutum* bears staple fibers 25–40 mm in length and 15 μm in thickness at their full maturity. Fiber cells must undergo four distinct but partially overlapped developmental stages, including initiation, elongation, secondary cell wall deposition, and maturation. The secondary cell wall of fiber, which is mainly composed of cellulose, is important especially for fiber quality perspective. However, some studies have shown that secondary cell wall of fibers of flax (*Linum usitatissimum* L.), ramie (*Boehmeria nivea* L.), and Spanish broom (*Spartium junceum* L.) also contain phenolics along with cellulose. Their fibers are known for their physical properties such as length and strength and have been used for textile purposes. A thicker secondary cell wall was estimated to contain no less than 70% cellulose content while the cotton fiber contains almost 90% cellulose [2, 3]. Lignin is another important component in cell wall [4]. It provides strength to plant cell wall and response to biotic and abiotic stresses in vascular plants [5]. The presence of lignin, which is reported at lower level in secondary cell wall of cotton fibers [6], negatively regulates fiber elongation and secondary cell wall synthesis in cotton. Studies demonstrated that the cotton plants that accumulate less lignin and lignin-like phenolics in mature fibers tend to have longer and stronger fibers [7]. From an active perspective, lignin and phenolics defend the cellulose fiber against dreadful conditions and increase the ability of response to biotic and abiotic stresses, and thus influence the growth and strength of plant cell walls [8]. Previous studies in herbaceous plants demonstrated the involvement of O-methyltransferases (*OMTs*) in lignin biosynthesis [9]. The involvement of *OMTs* mediate normal plant growth in the presence of lignin [10]. The initial *OMT* cDNA was described in 1991 [11], then a series of *OMT* cDNAs have been cloned from diverse plants species, including *Zea mays*, *Arabidopsis thaliana*, *Iris hollandica*, and *Nicotiana tabacum* [12].

According to substrate classification, plant methyltransferases have three major categories, I. O-methyltransferases (*OMTs*), II. N-methyltransferases (*NMTs*), and III. C-methyltransferases (*CMTs*). Category I *OMTs* are further classified into five sub-categories. Sub-category I-a comprises caffeoyl coenzyme A 3-O-methyltransferase (*CCoAOMT*) and caffeic acid 3-O-methyltransferases (*COMTs*), which are involved in methylation in phenylpropanoids. Sub-categories I-b, I-c, and I-d act in methylation of hydroxyl in flavonoid, alkaloids, and myoinositol, respectively. The fifth sub-category I-e takes part in methylation of carboxyl of diverse acids. The results of a study discovered the crystal structure of *OMTs* from *Medicago sativa* [13]. In the light of the explanations, the *OMT* gene that was cloned and characterized from a medicinal plant *Ligusticum chuanxiong* and contained higher ferulic acid was named as *LcCOMT*. The differential expression of *LcCOMT* gene under chilling stress was more than 6-fold higher than that under controlled conditions, suggesting that ferulic acid may increase plant tolerance to cold stress. BLAST analysis showed that *LcCOMP* was 23.9–40.2% similar to *OMTs* of alkaloid, flavonoid, isoflavonoid, and phenylpropanoids [14].

In the whole life cycle of cotton plant, it undergoes various environmental conditions from the cold spring in April when it is sowed to hot mid-summer when it grows rapidly in vegetation and reproduction and to late freezing autumn when it gets mature and is harvested. During the whole growth procedure, the cotton plant maintains an exquisite molecular controls and regulations. But little is known what roles the *OMT* family genes have played in cotton plant especially in early or late growth stage when season transition occurs, or in various stress conditions. Therefore, in this study, we identified the *OMT* family genes in the genome-wide scale and made detailed bioinformatics analysis of gene structure, chromosomal distribution, selection pressure during their evolution, sub-cellular localization, cis-regulatory elements etc., together with their expression profiling in different developmental stages and in responses to various stresses. Their expression profiling in developing fiber cells was verified using RNA sequencing data from RILs, CSSLs, and TM-1 at different fiber development stages. This study could open the way to comprehend the functions of *OMTs* in fiber quality advancement and in cotton plant responses to abiotic stresses, and thus could assume a noteworthy part for further investigation in the molecular mechanism of fiber improvement and stress tolerance.

## Results

### Genomewide identification and characterization of *OMT* genes

A genome wide analysis was conducted to characterize *OMT* family genes in three *Gossypium* species. A total of 192 *OMT* members were identified, including 82 in *G. hirsutum*, 55 in *G. arboreum*, and 55 in *G. raimondii* (Table S3. Sheet A). For phylogenetic analysis [15], 33 *OMT* members in *A. thaliana*, and 26 members in *T. cacao* species were also retrieved (Table S3. Sheet B). Retrieving information of *OMT* genes in *G. hirsutum* revealed that *GhOMT75_Scaf*, which was detected in scaffold, coded the smallest protein of 62 amino acids (aa) with a molecular weight of 6.642 kDa. While *GhOMT33_Dt*, which was identified on chromosome D_t_02, coded the largest protein of 969 aa with a molecular weight of 108.296 kDa among all *OMT* members in three *Gossypium* species.

In domain analysis of *OMT* family genes in *Gossypium* species, the results revealed that 64, 45 and 47 *OMTs* in *G. hirsutum*, *G. arboreum* and *G. raimondii* contained Pfam domain Pf00891, and that only 20, 10 and 9 *OMTs* in *G. hirsutum*, *G. arboreum* and *G. raimondii* contained Pfam domain Pf01596. In *A. thaliana* and *T. cacao*, 25, 24 OMTs contained Pf00891 domain, and 8, 2 OMTs contained Pf01596 domain respectively.

### Chromosomal distribution, collinearity, duplication, and loss of *OMT* genes

**Fig. 1:**
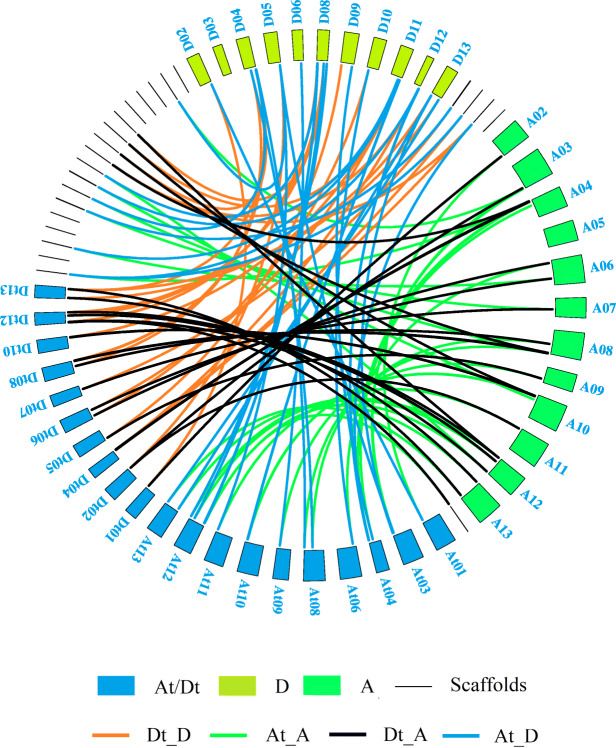
Collinearity analysis of *OMT* genes between A_t_D_t_ (*G. hirsutum*), A (*G. arboreum*), and D (*G. raimondii*) genomes

The analysis of chromosomal positioning was performed by using TBtools software [16]. A total of 161*OMT* genes were positioned on their respective chromosomes, while seven of *G. raimondii*, one of *G. arboreum*, and 23 of *G. hirsutum* were positioned in scaffolds (Figure S1). In *G. raimondii* (D genome), chr11 was mapped with 13 genes followed by chr08 with nine genes. The minimum number of genes in a chromosome was one in chr2, chr6, and chr10 respectively. There was no *OMT* family members identified in chr01 and chr07 (Figure S1.a). In *G. arboreum* (A-genome) (Figure S1.b), 54 *OMT* genes were mapped in all chromosomes except chr1. Chr10 harbored 13 *OMT* genes which were the highest per chromosome, followed by chr12 and chr04 with 10 and 9 genes respectively. The minimum number of genes located in a chromosome was one in chr02 and chr11 respectively. In *G. hirsutum* (A_t_D_t_ genome) (Figure S1.c), unexpectedly, there were no *OMT* genes in A_t_02, A_t_05, A_t_07, D_t_03, D_t_09, and D_t_11 chromosomes. The distribution of genes in D_t_ sub-genome (33 genes) was higher than in A_t_ sub-genome (26 genes). The maximum number of genes in a chromosome was seven in D_t_04 and A_t_12, followed by four in D_t_10 and A_t_10 chromosomes, respectively. D_t_01, D_t_05, A_t_01, A_t_06, and A_t_11 only had one *OMT* gene, and D_t_06, D_t_07, A_t_03, A_t_08, A_t_09, A_t_13 two *OMT* genes and D_t_02, D_t_08, and D_t_13 three *OMT* genes respectively (Figure S1.c). A collinearity analysis of the *OMT* family genes in *Gossypium* species chromosomes was shown in Fig. 1. The results demonstrated a pair wise collinearity of *OMT* genes between the chromosomes on which *OMT* family genes were mapped. Noticeably, a number of available genes in A_t_ and D_t_ scaffolds were collinear with their homologues in A and D genomes suggesting the collinearity of the DNA fragments between the scaffolds and chromosome where these *OMT* genes locate (Fig. 1). Taken the *OMT* gene numbers identified in each A/D genome or A_t_/D_t_ sub-genome, collinearity analysis also revealed that there were totally 21 and 19 *OMT* genes exclusively detected in A and D genomes respectively. Their homologous counterparts in A_t_D_t_ genomes of *G. hirsutum* are lost. There are also a few *OMT* genes that are exclusively detected in A_t_D_t_ genome of *G. hirsutum* without homologous counterparts in A and D genomes (Figure S1).

According to previous studies there are five types of duplications including singleton, dispersed, proximal, tandem, and segmental or whole-genome duplication [17]. In the present study, the analysis of gene pairs duplication events predicted a total of 31, 28, and 54 gene pairs of D_D_t_, A_A_t_ and D_A genomes from their common ancestor, 33 gene pairs of A_t__D_t_ subgenomes in segmental duplication, and 5 gene pairs of A_t__D_t_ subgenomes in tandem duplication events (Table S4 Sheet A).

### Analysis of selection pressure

In genetics, the Ka/Ks ratio used to estimate the balance between neutral mutations, purifying selections, and positive mutations based on a set of homologous genes [18]. The ratio of the number of non-synonymous substitutions per non-synonymous site (Ka) to the number of synonymous substitutions per synonymous site (Ks) represents selection pressure of the gene [19]. Ka/Ks < 1 demonstrates purifying selection pressure, while Ka/Ks = 1 and Ka/Ks > 1 show neutral and positive selection pressures respectively. Analysis of Ka/Ks ratio of homologous *OMTs* in three *Gossypium* species revealed that they are under purifying selection pressure. The Ka/Ks ratio of homologous *OMTs* in *G. raimondii* and *G. arboreum* ranged from 0.09 to 0.8, in *G. raimondii* and *G. hirsutum* ranged 0 to 0.7, and in A_t_ and D_t_ of *G. hirsutum* ranged 0.4 to 0.7 (Table S4 Sheet B).

### Sequences alignment, phylogenetic analyses, conserved motifs and gene structure

**Fig. 2:**
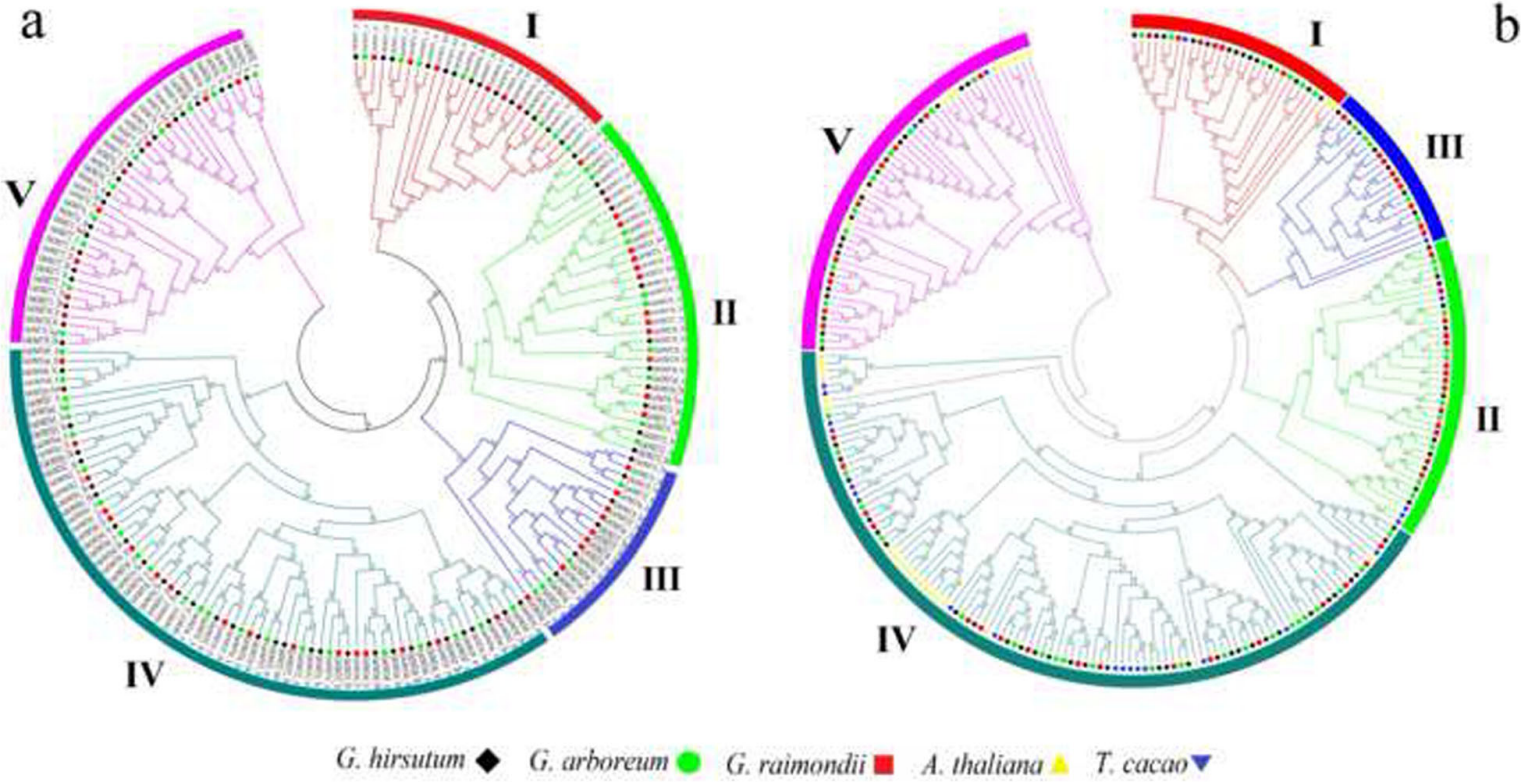
Phylogenetic analysis of *OMT* genes in *Gossypium*, *A. thaliana*, and *T. cacao* species. **a** Neighbor-joining phylogenetic tree of 192 *OMT* genes of *G. hirsutum*, *G. arboreum*, and *G. raimondii*. **b** Neighbor-joining phylogenetic tree of 251 *OMT* sequences of *G. hirsutum*, *G. arboreum*, *G. raimondii*, *A. thaliana*, and *T. cacao*. I, II, III, IV and V indicate the five groups of phylogenetic tree. Shapes with different colors represent *OMT* members of *G. hirsutum*, *G. arboreum*, *G. raimondii*, *A. thaliana*, and *T. cacao*

The sequence alignment of 251 *OMT* genes, including 192 genes from three *Gossypium* species, 33 from *A. thaliana*, and 26 from *T. cacao* species was performed to understand the phylogenetic relationship of these genes. The evolutionary relationship of *OMT* genes in three *Gossypium* species was monophyletic (Fig. 2a), and the member of *A. thaliana* and *T. cacao* were distributed in paraphyletic manner (Fig. 2b). According to the topology of constructed tree, the *OMT* gene family is divided into five clades (I, II, III, IV, and V) in *Gossypium*, *A. thaliana*, and *T. cacao* species. The results showed that each clade of *OMT* genes were symmetrically distributed within *Gossypium* species (Fig. 2a), while in *A. thaliana* and *T. cacao*, *OMT* genes were identified in cluster forms (Fig. 2b). The results demonstrated that these *Gossypium OMT* members might be evolutionary close within respective species and their identified clades.

To examine the conserved motifs of each clade, the analysis of representative motif logo and conserved motifs prediction were conducted (Figure S2). The results revealed that motif 1, enriched with leucine, valine, and glycine, motif 2, enriched with leucine and valine, and motif3, motif 4, motif 5, and motif 6 were common in clades I, II, III, IV and clade V. While motif 7 was found missing in some members of clade V, which was then replaced with motif 8 at same positions (Figure S2). The enriched amino acid residues of conserved motif1 (L/VDVGGG/TG) was previously identified in S-adenosyl-l-methionine (SAM)-dependant *OMTs* that shared 95% similarity with *G. hirsutum OMT* [20].

Investigation of gene structure has uncovered the different number of exons and introns of *OMT* genes. Exon and intron number of *OMT* genes varied from the least one exon and no intron to the most 7 to 9 exons and 6 to 8 introns (Table S5). Two members in *G. hirsutum* including *GhOMT82_At* and *GhOMT82_Dt* contain nine exons as the highest (Table S5). Same as, two members including *GaOMT82_A* in *G. arboreum*, and *GrOMT82_D* in *G. raimondii* contain 9 exons (Table S5). Gene structure analysis revealed that the *OMT* genes with higher number of exons had a shorter exons and introns, and vice versa. These results demonstrated that *OMT* members possess different structural patterns in accordance with their features.

### Identification of cis-regulatory elements in *OMT* family

The promoter regions of the *OMT* family contain precisely a large number of cis-regulatory elements. The analysis of cis-regulatory elements revealed the enrichment of MYB cis-regulatory elements, which was detected more than 350 times in *OMT* genes (Figure S3). The MYC was another important element that was found 183 times in enlisted *OMT* genes. Box 4 (part of a conserved DNA module involved in light responsiveness) was found 152 times in 43/82 genes in *G. hirsutum*. ABRE elements was detected 119 times in 29/82 *OMT* genes in *G. hirsutum*. The ERE element was detected 113 times in 37/82 and G-Box 97 times in 37/82 *G. hirsutum OMT* genes. An auxin RR-core and cis-acting regulatory element involved in the MeJA-responsiveness (TGACG-motif) were also observed in *Gossypium OMT* genes where this element was identified 48 times in 25/82 genes. Some other important cis-regulatory elements including wun-motif 44 times in 26/82, W-box 39 times in 31/82, GATA-motif 32 times in 27/82, O2-site 30 times in 22/82 *OMT* genes respectively, in *G. hirsutum* (Figure S3). These cis-regulatory elements might function collectively in accordance with their specific roles and with specific conditions as well as growth and development stages (Figure S3).

### Sub-cellular localization prediction of *OMT* genes

Understanding and determining the sub-cellular localization of proteins is an important strategy to identify the function of protein at cellular level [21]. This approach includes proteomic-based experiments and microscopic high throughputs [22, 23]. Several sequence-based approaches have been developed to predict the sub-cellular localization by providing amino acid sequences including PSORT [24], Yloc [25], BaCelLO [26], LOCtree [27]. According to CELLO prediction, most of *OMT* genes were located in the cytoplasm (Table 1), while seven genes were predicted in periplasm, including, *GhOMT45_At*, *GhOMT45_Dt*, *GhOMT46_Dt*, *GhOMT48_At*, *GhOMT48_Dt*, *GhOMT49_At*, and *GhOMT49_Dt*. Five *OMTs* were predicted to be localized in both periplasm and cytoplasm, including *GhOMT47_At*, *GhOMT47_Dt*, *GhOMT53_At*, *GhOMT54_Dt*, and *GhOMT68_At*. Two genes *GhOMT82_At* and *GhOMT82_Dt* were predicted in the outer membrane. Only *GhOMT55_At* was predicted in inner membrane and cytoplasm (Table. 1). The results of Wolf Psort were highly in agreement with those of CELLO analysis regarding the presence of most of the OMT genes in cytoplasm, however, with exceptions of *GhOMT48_At*, *GhOMT82_At*, *GhOMT82_Dt*, which were predicted in chloroplast and one gene *GhOMT76_Dt* in mitochondria (Table. 1). The function of the *OMT* genes might be related to their predicted localizations, though the experimental approach is still needed for further confirmation.

**Table 1:** Predicted Subcellular localization of *OMT* genes of *G. hirsutum*

Gene ID	CELLO	C_Reliability	Wolf Psort	P_Reliability	Gene ID	CELLO	C_Reliability	Wolf Psort	P_Reliability
GhOMT51_At	Cp	3.712	Cp	1	GhOMT10_Dt	Cp	3.384	Cp	6
GhOMT33_At	Cp	3.597	Cp	9.5	GhOMT7_Dt	Cp	4.717	Cp	11
GhOMT74_At	Cp	2.985	Cp	7	GhOMT8_Dt	Cp	4.717	Cp	7
GhOMT40_At	Cp	3.897	Cp	12	GhOMT9_Dt	Cp	4.735	Cp	9
GhOMT68_At	Pp/Cp	2.094/2.624	Cp	6	GhOMT79_Dt	Cp	3.973	Cp	10
GhOMT71_At	Cp	4.545	Cp	5	GhOMT81_Dt	Cp	3.924	Cp	6.5
GhOMT78_At	Cp	4.122	Cp	13.5	GhOMT78_Dt	Cp	2.531	Cp	2
GhOMT81_At	Cp	2.707	Cp	5	GhOMT54_Dt	Pp/Cp	1.967/2.566	Cp	8
GhOMT3_At	Cp	4.033	Cp	2	GhOMT76_Dt	Cp	3.273	Mc	8
GhOMT10_At	Cp	4.822	Cp	6	GhOMT32_Dt	Cp	4.607	Cp	12
GhOMT12_At	Cp	4.154	Cp	4	GhOMT30_Dt	Cp	3.946	Cp	1
GhOMT6_At	Cp	4.234	Cp	8	GhOMT31_Dt	Cp	4.695	Cp	6
GhOMT76_At	Cp	4.275	Cp	11.5	GhOMT49_Dt	Pp	3.275	Cp	8
GhOMT32_At	Cp	4.675	Cp	8	GhOMT57_Dt	Cp	4.391	Cp	7
GhOMT30_At	Cp	4.743	Cp	6	GhOMT77_Dt	Cp	2.352	Cp	3
GhOMT49_At	Pp	3.107	Pp	11	GhOMT58_Dt	Cp	4.675	Cp	5
GhOMT77_At	Cp	3.838	Cp	7	GhOMT63_Dt	Cp	3.572	Cp	10
GhOMT57_At	Cp	2.229	Cp	9	GhOMT62_Dt	Cp	2.968	Cp	4
GhOMT52_At	Cp	1.577	Cp	10	GhOMT61_Dt	Cp	3.224	Cp	4
GhOMT53_At	Pp/Cp	1.995/1.743	Cp	6.5	GhOMT17_Dt	Cp	4.112	Cp	2
GhOMT62_At	Cp	3.577	Cp	2	GhOMT13_Dt	Cp	4.124	Cp	7
GhOMT65_At	Cp	3.508	Cp	8	GhOMT70_Dt	Cp	4.385	Cp	13
GhOMT24_At	Cp	4.529	Cp	8	GhOMT1_Dt	Cp	4.927	Cp	10
GhOMT14_At	Cp	4.404	Cp	12	GhOMT55_Dt	Cp	2.781	Cp	1
GhOMT29_At	Cp	4.069	Cp	1	GhOMT82_Dt	OM	2.226	Chp	9
GhOMT41_At	Cp	4.655	Cp	6	GhOMT45_Dt	Pp	3.910	Cp	6
GhOMT70_At	Cp	3.408	Cp	8	GhOMT46_Dt	Pp	2.490	Cp	10
GhOMT1_At	Cp	4.921	Cp	7	GhOMT47_Dt	Pp/Cp	1.961/2.316	Cp	10
GhOMT55_At	IM/Cp	1.807/2.478	Cp	1	GhOMT48_Dt	Pp	4.276	Cp	8
GhOMT82_At	OM	2.374	Chp	9.5	GhOMT72_Dt	Cp	4.474	Cp	11.5
GhOMT45_At	Pp	4.340	Cp	7	GhOMT60_Dt	Cp	4.312	Cp	7
GhOMT47_At	Pp/Cp	2.121/2.358	Cp	12	GhOMT59_Dt	Cp	4.534	Cp	6
GhOMT48_At	Pp	4.154	Chp	6	GhOMT5_Scaf	Cp	4.641	Cp	11
GhOMT2_At	Cp	4.870	Cp	5	GhOMT11_Scaf	Cp	4.885	Cp	4
GhOMT72_At	Cp	4.493	Cp	13.5	GhOMT68_Scaf	Cp	3.973	Cp	8
GhOMT60_At	Cp	4.637	Cp	5	GhOMT52_Scaf	Cp	2.277	Cp	4
GhOMT51_Dt	Cp	3.636	Cp	2	GhOMT46_Scaf	Cp	2.154	Cp	11
GhOMT37_Dt	Cp	4.092	Cp	6	GhOMT15_Scaf	Cp	3.576	Cp	9
GhOMT35_Dt	Cp	4.228	Cp	4	GhOMT75_Scaf	Cp	2.090	Cp	5
GhOMT33_Dt	Cp	3.130	Cp	8	GhOMT73_Scaf	Cp	3.123	Cp	6
GhOMT71_Dt	Cp	4.475	Cp	11.5					
GhOMT6_Dt	Cp	4.619	Cp	8					

Cp: Cytoplasmic, Pp: Periplasmic, OM: outer membrane, IM: inner membrane, Chp: Chloroplast, Mc: Mitochondria

C_Reliability: Lower reliability values show the stronger possibility of predicted localization

P_Reliability: Higher reliability values show the stronger possibility of predicted localization

### GO enrichment and KEGG pathway analyses

**Fig. 3:**
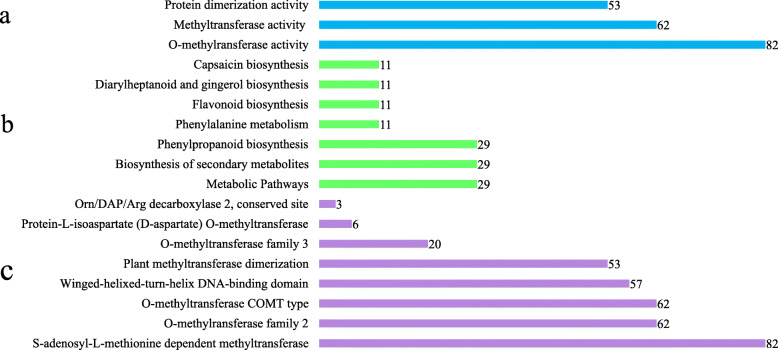
Enrichment analysis of *OMT* family genes in *G. hirsutum*. **a** The functional annotations of Gene Ontology. **b** The functional annotations of KEGG pathways. **c** The functional annotations of Interpro. The scales indicate the enriched gene number in respective categories

To understand the functional annotations of *OMT* family genes of *G.hirsutum*, 82 genes in *G. hirsutum* were undergone through gene ontology (GO) enrichment, kyoto encyclopedia of genes and genomes (KEGG Pathway), and InterPro analyses. GO term analysis verified their O-methyltransferase activity of all 82 *OMT* genes, while 62 of the 82 genes were also enriched in methyltransferase activity and 53 of the 82 genes in protein dimerization activity (Fig. 3a). KEGG Pathway analysis revealed that these *OMTs* were involved in different metabolic pathways. Twenty-nine *OMTs* were involved in monolignol biosynthesis, phenylpropanoid, secondary metabolism, and metabolic pathways respectively. Eleven genes were involved in phenylalanine and flavonoid biosynthesis pathways respectively (Fig. 3b). InterPro analysis (http://www.ebi.ac.uk/interpro/) categorized these 82 *OMT* genes as functional genes of S-adenosyl-L-methionine-dependent methyltransferase (Fig. 3c). Sixty-two genes were also predicted in categories of methyltransferase_2 and O-methyltransferase COMT-type respectively (Fig. 3c), while fifty-seven in winged helix-turn-helix DNA-binding domain, fifty-three in plant methyltransferase dimerization (Fig. 3c).

### Expression profiling of *OMT* genes and their homologues in fiber development and salt stress

**Fig. 4:**
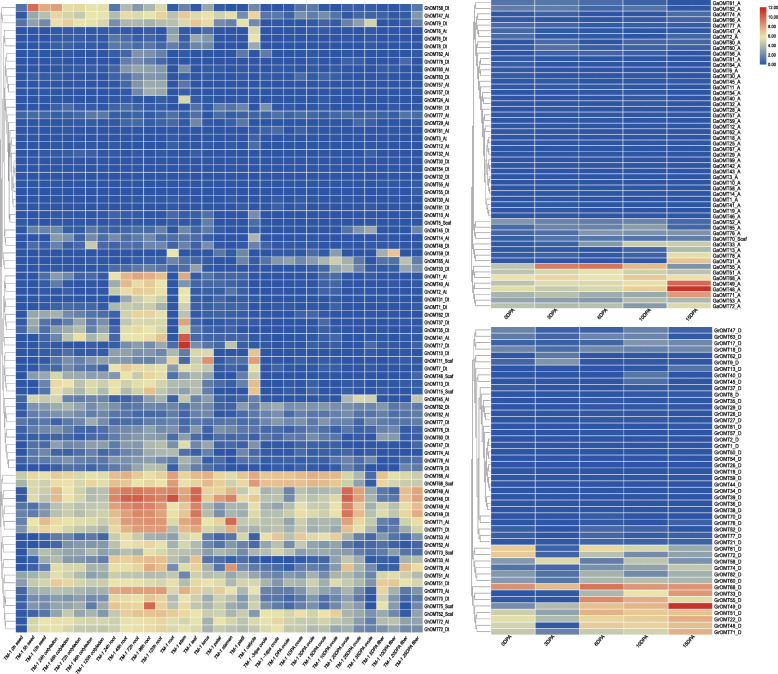
Transcriptome analysis of *OMT* family genes in different growth, ovule and fiber development stages. **a**: Transcriptome heatmap of *GhOMT* family genes in standard genetic cultivar TM-1 of *G. hirsutum* at different growth, ovule and fiber development stages [28, 31]. **b**: Transcriptome heatmap of *GaOMT* family genes in *G. arboreum* [31]. **c**: Transcriptome heatmap of *GrOMT* family genes in *G. raimondii* [32]

In order to verify the biological functions of *OMT* family genes, several transcriptome data sets including TM-1 [28], *G. arboreum*, *G. raimondii*, CSSLs [29], and RILs [30], were applied to analyze their expression profiles in different developmental stages, organs, or tissues, and responses to various abiotic stress treatments. The transcriptome clusters showed that the *OMT* genes can be assorted into three basic groups (Fig. 4a): Those that have a broad responses to different developmental stages from germination to fiber maturation, typical examples of which included *GhOMT48_At*, *GhOMT48_Dt*, *GhOMT49_At* and *GhOMT49_Dt*; those that have specific responses to root development, including *GhOMT1_At*, *GhOMT2-At* and *GhOMT40_At*; and those that have responses to early germination in seed, cotyledon, root and stem, including *GhOMT47_At*, *GhOMT9_Dt*, and *GhOMT58_Dt*. When fiber specific transcriptome data sets of *G. arboreum*, *G. raimondii* were applied to observe the expression profiling diploid *OMT* family genes, the result also supported specific expression profiling of some *OMT* genes in diploid species of *G. arboreum* (Fig. 4b) and *G. raimondii* (Fig. 4c).

**Fig. 5:**
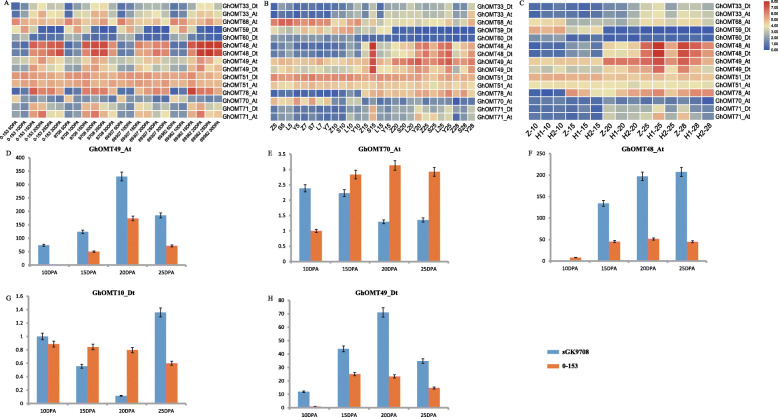
Specific responses of selected *OMT* genes in fiber development. **a** Transcriptome heatmap of selected *GhOMT* genes in RIL lines and their parents [30]. **b** Transcriptome heatmap of selected *GhOMT* genes in CSSLs of CCRI45 and Hai1 [33, 34]; Z, S, L, and Y represent CCRI45, MBI7561, MBI7747, and MBI7285, respectively; 5, 7, 10, 15, 20, 25, and 28 represent different DPA. **c** Transcriptome heatmap of selected *GhOMT* genes in CSSLs of CCRI36 and Hai1 [29, 34]; Z, H1, and H2 represent CCRI36, MBI9915, and MBI9749; 10, 15, 20, 25, and 28 represent different DPA. **d-h** qRT-PCR verification results of *GhOMT49_At*, *GhOMT70_At*, *GhOMT48_At*, *GhOMT10_Dt*, *GhOMT49_Dt* in developing fibers of sGK9708 and 0–153 at 10, 15, 20, and 25 DPAs

The gene expression profiling was further verified with trancriptome datasets of RILs (Fig. 5a) two CSSLs (Fig. 5b and c). The results showed that the genes that had specific expressions during fiber development (Fig. 4a) also had specific expressions in fiber development of RILs and CSSLs materials. These genes had a highly consistent expression profiling among the different cotton cultivars and lines during fiber development. Some selected *GhOMT* examples genes, *GhOMT49_At* (Fig. 5d), *GhOMT70_At* (Fig. 5e), *GhOMT48_At* (Fig. 5f), *GhOMT10_Dt* (Fig. 5g), and *GhOMT49_Dt* (Fig. 5h), were verified through qRT-PCR using sGK9708 and 0–153, the two parental lines of the RIL population with different fiber quality traits. The results showed that *GhOMT48_At*, *GhOMT49_At*, and *GhOMT49_Dt* were significantly up-regulated during fiber development in sGK9708 than in 0–153 Fig. 5d, f and h) and that *GhOMT70_At* and *GhOMT10_Dt* did not show differences between the two cultivars (Fig. 5e and g). Noticeably, *GhOMT49_At* and *GhOMT49_Dt* reached the highest expression levels at 20 DPA and their high expression lasted in a short time as compared with that of *GhOMT48_At*. *GhOMT48_At* had a rapid expression increase from 10 DPA to 15DPA and then its expression steadily increased until 25 DPA when it reached its highest expression level.

**Fig. 6:**
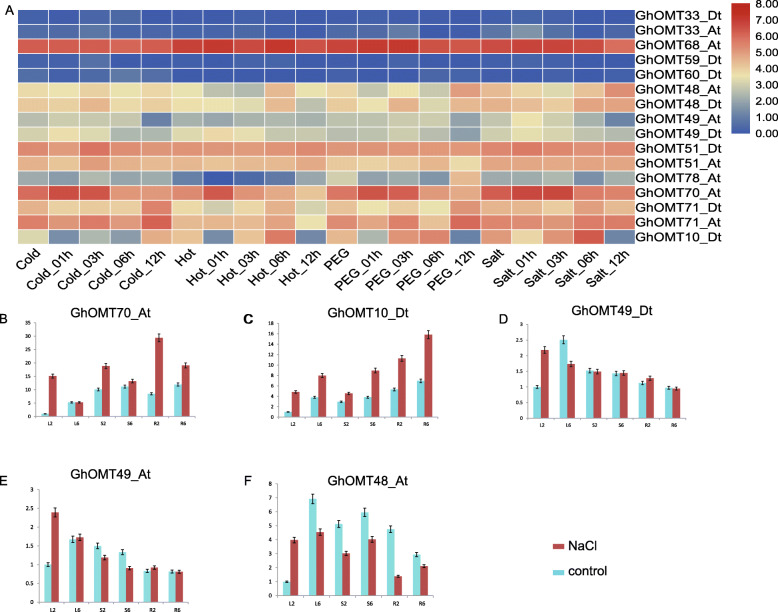
Specific responses of selected *OMTs* in salt stress treatment. **a** Transcriptome heatmap of selected *GhOMT* genes in cold, hot, osmotic and salt treatments [28, 31]. **b-f** qRT-PCR verification results of *GhOMT70_At*, *GhOMT10_Dt, GhOMT49_Dt*, *GhOMT49_At*, and *GhOMT48_At* in salt treatments of sGK9708

Based on the expression profiling of the *OMT* gene family in responses to cold, hot, osmotic, and salt stress treatments (Fig. 6a), two genes specific in salt stress responses, *GhOMT70_At* and *GhOMT10_Dt*, and three genes specific in fiber development, *GhOMT48_At*, *GhOMT49_At*, and *GhOMT49_Dt*, were verified by qRT-PCR with RNA samples extracted from salt treatment. The results indicated that both *GhOMT70_At* and *GhOMT10_Dt* showed an elevated expression in salt treatments in salt-tolerant cultivar as compare to the control treatments (Fig. 6b and c). These two genes had different expression profiles from 2 h to 6 h after salt treatment. *GhOMT70_At* had the highest expression at 2 h and then its expression went down at 6 h; whereas *GhOMT10_Dt* had an increasing expression pattern from 2 h to 6 h. Both genes had much higher expression in roots than in stem or leaf.

## Discussion

### A genome-wide survey of *OMTs*

A genome wide search of *G. hirsutum* [28], *G. arboreum* [35], and *G. raimondii* [36] resulted in the identification of 192 genes (82 in *G. hirsutum*, 55 in *G. arboreum*, and 55 in *G. raimondii*). Recent study testified that modern allotetraploid *Gossypium* species were developed from a natural hybridization between the ancestors of two diploid species of *G. raimondii* (D-genome) [32] and *G. arboreum* (A-genome) [35] 1.7 to 1.9 million years ago [28]. The results of current study revealed a loss of quite a large number of *OMT* genes in *G. hirsutum* A_t_D_t_ genome as compared to the total number of *OMT* genes in A and D genomes. Possibly 19 *OMT* genes in A_t_ sub-genome and 17 in D_t_ sub-genome in *G. hirsutum* (Figure S1) were lost during the evolution procedure after it arose from above mentioned hybridization [28]. Gene losses can be the result of premature stop codon, disruption of genes as compared to their orthologous [37], and rapid genome re-organization during polyploidization and diploidization process [38–40]. Previous studies have evidenced that polyploidization processes may result in losing of homologous members or altered expression profiles of the homologous genes or both [31, 41–43]. Similar phenomenon was noticed in the expression profiling of homologous *OMT* genes between A_t_ and D_t_ in *G. hirsutum*, which clued that these genes might have experienced abovementioned events during evolution processes. Collectively, a higher number of genes were also identified in whole genome duplication event. The whole genome duplication may have resulted from an organism that inherited two genomes from each parent. Whole genome duplication events results duplicate genes that may lost through fractionation [44]. Besides the whole genome duplication, segmental duplication events were also identified with a large number in *OMT* gene pairs. Segmental duplication is widespread in flowering plants, which might lead to the evolution of novel genes and their functions [45].

Phylogenetic analysis showed high similarity and monophyletic distribution of *OMTs* within *Gossypium* species that might support the conservative evolution mode of *OMT* genes within five phylogenetic clades. Previous study also reported five clades of *OMT* genes in *Catalpa bungei* [46]. Analyses of selection pressures revealed that most of *OMT* genes in *Gossypium* species were under a purifying selection pressure. The purifying selection pressure might suggest the importance of *OMT* genes in *Gossypium* species. But noticeable exceptions were also observed in some interspecific homologous pairs, in which their Ka/Ks values were > 1, indicating these homologous pairs were under a positive selection pressure. These homologous pair exceptions included *GrOMT52_D*-*GhOMT52_Scaf* and *GrOMT29_D*-*GhOMT29_At* in *G. raimondii* and *G. hirsutum*, *GaOMT30_A*-*GhOMT30_At* in *G. arboreum* and *G. hirsutum*, *GrOMT63_D*-*GaOMT64_A* and *GrOMT29_D*-*GaOMT29_A* in *G. raimondii* and *G. arboreum*. These results suggested that the *OMT* genes might had experience positive selection pressures during the evolution from diploids to tetraploids. Previous studies have evidenced that the positive selection pressure might be associated with the onsets of new functions in genes [47, 48]. Considering the fact that quite a proportion of *OMT* genes were lost during the formation and evolution of allotetraploid cotton (see afore discussion and Figure S1). In the current study, six *OMT* family members in *G. hirsutum*, one in *G. arboreum* and nine in *G. raimondii* were characterized as (R,S)-reticuline 7-O-methyltransferase. *7OMTs* convert reticuline to laudanine in tetrahydrobenzylisoquinoline biosynthesis in the opium poppy *Papaver somniferum*, however, this enzymatic activity is unknown in most higher plants [49]. Therefore, how these genes function is still open to discussion. Taken all findings together, the results might suggest that the *OMTs* that experienced positive selective pressure be lost or take on some novel functions in *G. hirsutum* during the processes of its evolution and ancestor formation.

Previous findings have reported that the *G. raimondii* (D-genome) and *G. arboreum* (A-genome) are the closest relatives to the D_t_ and A_t_ sub-genomes of allotetraploids, respectively [28]. Each gene in A or D genome will always have a homolog in the correspondent A_t_ or D_t_ sub-genomes of *G. hirsutum* [50]. However, in both A and D genomes we detected quite a large number of *OMT* genes that do not have homologs in their relative A_t_ and D_t_ sub-genomes (Figure S1). Previous studies evidenced that such homolog loss could result from two possible reasons: one is that the homologs were lost during the procedure of polyploidization from diploids to tetraploid; the other is that after the tetraploid formation, the *OMT* members in each genome started their separate evolution procedure. This separate evolution procedure makes the newly evolved members have no homologs in its relative genomes [28]. Previous studies revealed that in A, D, A_t_D_t_ genomes do not maintain same speed of evolution. A faster evolution rate was observed in allotetraploid cottons than in diploid cottons [28]. Taken the fact that *OMT* genes undergo purifying selection procedures (Table S4. Sheet B), the first reason is possibly endorsed as the main cause for the current evolution status of *OMT* gene family and the second reason may also play a role.

### Function prediction of *OMT* candidates

#### *OMTs* are involved in diverse cis-regulatory elements

Plants encounter various biotic and abiotic stresses during their entire life cycles that negatively affect growth, development, and productivity [51]. Under exposure of these stresses, plants require some potential mechanism, which can be activated in critical circumstances, to support whole plant life cycle [52]. Excessive salinity is also a major factor that affects the cotton production all around the world [53]. Identification of cis-regulatory elements revealed that the *OMT* genes are enriched with important cis-regulatory elements that are essential against negative environmental stresses. Some important regulatory elements, including W-box, MYB, MYC, DRE, ABRE, G-Box, MBS [54], were identified in *OMT* genes. W-box is important to regulate the expression of genes and to bind *WRKY TFs*. *WRKY TFs* are important to mediate plants to defense against chilling, wounding, drought, salinity and heat stresses [55–63]. MYB and MYC have been identified as involved in dehydration-response [64]. DRE [65], which up-regulate gene expression under cold stress and increase the tolerance of plants was also identified in these specific genes. ABRE is an important regulatory element that enhances salt stress tolerance in plants. It plays a key role in dehydration and in response to salinity stress in *Arabidopsis thaliana*, soybean and rice, and in response to chilling or cold in *Paeonia suffruticosa* [66]. G-box is identified in several gene promoters in previous studies and it contributes to development, hormone response, and tolerance against fungal infections in plants. Besides, a gibberellins response element (GARE) was also identified to be important to promote flowering in plants. The auxin hormones play a major role in growth and development of diverse plant species [67]. These results were in accordance with our findings. Especially the repetitively identified cis-regulatory elements might have biological functions in plants under specific conditions and development stages.

#### OMTs are possibly involved in secondary metabolic pathways

The KEGG pathways enrichment analysis revealed the involvement of *OMT* genes in secondary metabolism and metabolic pathways including monolignol, phenylpropanoid, flavonoid, and phenylalanine metabolisms. Secondary metabolic pathways are demonstrated to have exceptional impacts on biotic and abiotic stresses. Secondary metabolites are phytochemicals, which are synthesized through secondary metabolism. In plants, phenylpropanoids are categorized in several groups such as phenolic acids, flavonoids, and lignins, which are involved in diverse physiological processes and tolerance under unfavorable conditions [68–72]. The activity of secondary metabolites increases during the response of abiotic stresses. These phenolics provide plants with higher tolerance against heavy metals [73, 74], salinity [75], drought [76], and temperature stresses [71]. These pathways also play an important role in plant cell elongations [77, 78]. Same as, plant *OMT* genes have been identified in secondary metabolism [79]. Higher expression of secondary metabolic pathways related genes in developing cotton fiber is reported in previous studies [80, 81]. Importantly, *OMT* genes were reported to be involved in lignin synthesis and to be induced by inoculation of *Verticillium dahliae* in cotton [82–84]. During the inoculation of pathogens, changes in the expression patterns of phenylpropanoid related *OMT* genes were identified. These identified *OMT* genes included *GhOMT53_At*, *GhOMT58_Dt*, *GhOMT61_Dt*, and *GhOMT78_Dt* that were found significantly expressed in 12 and 48 h post inoculation *V. dahliae* [85]. In the current study, these genes were down-regulated under abiotic stresses and in fiber development stages (Fig. 4). Previous reports have evidenced that desoxyhemigossypol-6-O-methyltransferase (dHG-6-OMT) catalyzed the biosynthesis of terpenoid and provided an effective defense mechanism to cotton plant against biotic stresses including insects and pathogens [86]. In response to *V. dahliae* (V991) in CSSLs lines CCRI36 and MBI8255, diverse genes were found differentially expressed in lignin biosynthesis including *CCoAOMT*, which can adequately utilize lignin and has been characterized in several previous studies [87, 88]. Another study also reported that *CCoAOMT* was up-regulated in response to *Verticillium* pathogen in cotton and rendered cotton plants a comparable phenotypic resistance as compared to control plants [89]. A RNA-seq analysis based research identified differential expression patterns of *CCoAOMT* in response to *V. dahliae*, confirming the effect of this *OMT* gene in the plant response to *V. dahliae* in cotton [90]. These results consequently evidenced the important role of secondary metabolic pathways and *OMT* genes in biotic stresses in cotton.

#### OMTs are possibly involved in plant growth, abiotic stress tolerance, and fiber development of cotton

Salinity is one of the major causes to reduce crop yield [91] and incurs up-regulation and/or down-regulation of plant genes in response [92]. The *OMT* genes have been found specific for salt stress tolerance and fruit development in tomato plant (*Solanum lycopersicum*) [93]. The SAM-dependent methyltransferases genes were identified to play important role in sweet potato (*Ipomoea batatas*) in response to salt stress [94]. In wheat, *TaCOMT-3D* contributes to stem mechanical support [95]. Another *TaCOMT* gene was also observed with constitutive expression in stem along with leaf and root [96]. The *OMT* gene (*BdCOMT1*) was strongly expressed in stem node and internode but poorly expressed in other tissues in *Brachypodium distachyon* plant [97]. The expression profiles of *OMT* gene family in the transcriptome data of TM-1 [28] and verification results through qRT-PCR also suggested that two *OMT* members *GhOMT10_Dt* and *GhOMT70_At* might contribute to salt stress tolerance in *G. hirsutum*. In the qRT-PCR verifications, *GhOMT10_Dt* and *GhOMT70_At* showed different expression profiling from 2 h and 6 h after 200 mM NaCl treatment (Fig. 5). Probably they act differently in response to salt stress in *G. hirsutum*. Five genes including *GhOMT1_At*, *GhOMT41_At*, *GhOMT47_At*, *GhOMT17_Dt*, and *GhOMT37_Dt* had significant expressions in stem (Fig. 4a) where they might be the potential candidates to provide structural support and survival to plant in environmental stresses.

Cotton fiber quality of is an important attribute to develop elite cultivars in the presence of negative environmental factors. Studies demonstrated that *GhOMT48_At* and *GhOMT49_At* were expressed at elongation stages of a CSSL (CS-B25) and TM-1 respectively [28, 98]. In the current study, the fiber specific *OMT* genes were consistently identified across various populations and species including TM-1 (Fig. 4a) *G. arboreum* (Fig. 4b) *G. raimondii* (Fig. 4c), RILs (Fig. 5a), CSSLs (Fig. 5b, c). They also showed highly similar expression patterns in different fiber development stages. The expression specificities of *GhOMT48_At*, *GhOMT49_At*, and *GhOMT49_Dt* in developing fibers were further verified through qRT-PCR studies (Fig. 5d, f and h). The results demonstrated that these *OMT* members could have a significant function in fiber development and fiber quality formation. But how these genes function during fiber quality formation was still open to discussion.

Lignins-like phenolics are widely studied in response to stress [99]. Recent research advancements revealed that lignin or phenolics influence fiber development at elongation and secondary cell wall synthesis stages [100]. The knock-down of Lignin-like phenolics related gene (*GhbHLH18*) in *G. hirsutum* evidenced the regulation of lignin-like phenolics pathway genes including a *COMT* and others, during cotton fiber elongation and secondary cell wall synthesis stages. The results demonstrated the roles of these genes in regulating the lignification in developing cotton fibers [7]. This study has gathered important information of *OMT* gene family which is a forward step in research to uncover the possible functions or to support previous studies in exploration the functions of *OMT* genes in plant response to salt stress and in cotton fiber development.

## Conclusions

Methyltransferases are versatile class of enzymes. *OMT* contributes to diverse phenolics that are essential for plant growth and serves as protective shield against several kinds of stresses. Various bioinformatics analyses revealed that *OMT* gene family is a strong growth regulator, which not only provide protection to the plant, but also are involved in fiber elongation and secondary cell wall synthesis stages. Furthermore, expression profiling analysis based on several transcriptome data and qRT-PCR validation inferred that *GhOMT10_Dt* and *GhOMT70_At* might be the potential candidates for salt stress tolerance and that *GhOMT48_At*, *GhOMT49_At*, and *GhOMT49_Dt* might have significant influence in fiber development at elongation and secondary cell wall thickness stages of *G. hirsutum*. This proposed study concludes the important roles of *OMT* family genes in cotton fiber development and in salt stress tolerance.

## Methods

### Identification of *OMT* protein family members, sequences alignment, and phylogenetic tree construction

Genome data of three *Gossypium* species including *G. arboreum* (CRI), *G. raimondii* (JGI), and *G. hirsutum* (NAU) were downloaded from cotton functional genomic database (https://cottonfgd.org/) [101]. Genome data of *A. thaliana* (Athaliana/TAIR10, https://genome.jgi.doe.gov/portal/pages/dynamicOrganismDownload.jsf?organism=Athaliana#) [102] and *T. cacao* (Tcacao/v2.1, https://genome.jgi.doe.gov/portal/pages/dynamicOrganismDownload.jsf?organism=Tcacao#) [103] were also downloaded for comparative analysis of *OMT* genes. The hidden Markov model profiles (PF00891 and PF01596) were downloaded from Pfam database (https://pfam.xfam.org/). The hmmsearch program of HMMER 3.0 software [104] was used to search for protein sequences of three *Gossypium* species with the E-value of 1e− 5. OMT protein sequences of *A. thaliana* and *T. cacao* were also retrieved from the Phytozome database (https://phytozome.jgi.doe.gov/pz/portal.html) for phylogenetic analysis. The proteins with absence of required domains were manually removed. Other features of *OMT* genes including protein length (aa), and molecular weight (kDa) were characterized by using cotton functional genomic database (http://www.cottonfgd.org/) [101]. The full length amino acid sequences of *G. hirsutum*, *G. arboreum*, *G. raimondii*, *A. thaliana*, and *T. cacao* encoded by *OMT* genes were aligned with clustalx2 software (http://www.clustal.org/) [105] with default parameters for the neighbor-joining phylogenetic tree as 1000 bootstraps. Subsequently, two neighbor-joining phylogenetic trees were generated by using Mega7 [106]. The topology of both phylogenetic trees was confirmed to understand the phylogenic relationship within the five plant species.

Nomenclature of these members was based on their chromosomal locations, homology and numbers in each *Gossypium* species.

### Chromosomal mapping and collinearity analysis

TBtools was used to perform the chromosomal mapping of the given *OMT* genes, to search the homologous pairs of *OMT* genes between genomes of the three *Gossypium* species through protein-protein blast (E-value le-5). Circle gene viewer model of TBtools software was used to visualize the results of collinearity between homologous gene pairs [16].

### Gene structure and conserved motifs

The structure of the *OMT* genes was analyzed using the online server of Gene Structure Display (GSDS 2.0, http://gsds.cbi.pku.edu.cn) [107]. The conserved motifs were predicted online in MEME web based motif prediction tool version 5.0.5 (http://meme-suite.org/) by providing protein sequences of *OMT* genes [108].

### Selection pressure, cis-regulatory elements, sub-cellular localization and gene enrichment analysis

The CDS of homologous gene pairs of *G. hirsutum* (NAU), *G. arboreum* (CRI), and *G. raimondii* (JGI) were assigned to TBtools software to estimate the Ka/Ks ratio to predict selection pressure between the genes of each pair in genomes and sub-genomes [16]. The upstream sequences (2000 bp) of *OMT* genes were retrieved through cotton functional genomic database (http://www.cottonfgd.org) and were submitted to PlantCARE database [109] to obtain the cis-regulatory elements. Sub-cellular localization of genes was predicted using online bioinformatics tools CELLO v.2.5 and Wolf Psort with their protein sequences [24, 110]. KEGG IDs of *OMT* family genes were downloaded from cotton functional genomic database (http://www.cottonfgd.org), then annotation was performed by providing KEGG IDs in kyoto encyclopedia of genes and genomes database (https://www.genome.jp/kegg/) [111]. Gene ontology (GO) annotation IDs of *OMT* family genes were downloaded from cotton functional genomic database (http://www.cottonfgd.org) and were submitted to Gene ontology database (http://geneontology.org/) to perform GO analysis [112].

### Expression profiling of *OMT genes*

The different sets of RNA sequencing data including TM-1, a genetic standard line of *G. hirsutum* (Nanjing Agricultural University, Nanjing, Jiangsu, China) (PRJNA248163) [28, 31], 69,307 and 69,362 (selected lines from a RIL population sGK9708 × 0–153, Institute of Cotton Research, Anyang, Henan, China) (PRJNA542946) [30], MBI7747, MBI7561, and MBI7285 (selected lines from CSSL population CCRI45 × Hai1, SRP084203) [33, 34], and MBI9915 and MBI9749 (selected lines from CSSL population CCRI36 × Hai1, SRX2843778) (Institute of Cotton Research, Anyang, Henan, China) [29, 34] were included in this study to observe the expression pattern of *OMT* family genes at different growth stages, under abiotic stress treatment stages, ovule development, and in different fiber development stages of cotton. Briefly, 69,307, 0–153, MBI7747, MBI7561, MBI9915, MBI9749, and Hai1 have high fiber quality traits, while 69,362, MBI7285, sGK9708, CCRI36, and CCRI45 have low fiber quality traits. Detailed information of these referenced materials is presented in Table S1.

Transcriptome data of *G. arboreum* (PRJNA179447) [35], and *G. raimondii* (PRJNA79005) [32] were also included to compare the comparative expression of these *OMT* genes.

### Plant material, RNA isolation, cDNA synthesis, and qRT-PCR

Upland cotton cultivar 0–153 had elite fiber quality while sGK9708 had high yield potential and wide adaptability. They are successfully used to tag fiber quality and yield QTLs in our previous reports [30, 113, 114]. In the current study, sGK9708 and 0–153 (Table S1) were planted in April 2018 in the experimental fields of the Institute of Cotton Research, Chinese Academy of Agricultural Sciences, Anyang, Henan. Flowers were tagged on the day of anthesis for fiber sampling in July 2018. Bolls of tagged flowers were sampled in the morning between 9:00 and 10:00 AM at 10, 15, 20, and 25 days’ post anthesis (DPA). The fibers were dissected from the developing seeds right after boll picking and immediately stored at − 80 °C for RNA extraction.

To examine the expression profiling of *OMT* genes under salt stress, seeds of sGK9708 cultivar were germinated in wet filter papers for 72 h and then were transferred to hydroponic conditions. The seedlings were treated with 200 mM NaCl at three leaves stage. The true leaves, stems, and roots were sampled at 0 h, 2 h, and 6 h of the treatment. The 0 h of treatment was considered as control sample to compare the expression profiling with treated samples.

Total RNA isolation was performed with the RNAprep Pure Plant Kit by (Tiangen, Beijing, China). To eliminate the genomic DNA contamination, the RNA samples were treated with DNase1. RNA concentration and integrity was observed on Nano Drop 2000 spectrophotometer (Thermo scientific, USA) and 1% agarose gel electrophoresis. cDNAs of the RNA samples that the A260/280 ratio reached 2.00 were synthesized using PrimeScript® RT Reagent Kit (Perfect Real Time, Takara Biotechnology Co., Ltd., Dalian, China). qRT-PCR was performed with ABI 7500 fast Real-Time PCR system (Applied Biosystems, USA), with *Gh-Histone3* gene was used as reference to normalize the relative expression level. Primers pairs of five *OMT* genes were designed by using Oligo 7 [115] (Table S2). 2^−ΔΔ^Ct method was used to calculate the gene expressions [116].

## Supplementary Information

**Additional file 1: Figure S1.** Chromosomal distribution of OMT genes in the genomes of Gossypium species. a: in D genome (*G. raimondii*), b: in A genome (*G. arboreum*), c:in A_t_D_t_ genomes (*G. hirsutum*), d: in scaffolds of the three genomes. The genes in boxes in A and D genomes represent that their homologous genes in A_t_ and D_t_ sub-genomes are missing, while the genes in boxes in At and Dt sub-genomes represent that their homologous genes in A and D genomes are missing.

**Additional file 2: Figure S2.** Identification of motifs of OMT genes in three Gossypium species.

**Additional file 3: Figure S3.** Identification of cis-regulatory elements of OMT genes.

**Additional file 4: Table S1.** Detail Information of plant materials used for RNA-seq data acquisition.

**Additional file 5: Table S2.** Sequences of Primer Pairs of five selected *OMT* family genes for qRT-PCR verification.

**Additional file 6: Table S3.** Basic information and analyses of OMT genes in *Gossypium* species (Sheet A), and in *A.* *thaliana* and *T. cacao* (Sheet B).

**Additional file 7: Table S4.** Analyses of duplication events and selection pressure. Sheet A: The analysis of duplication events of OMT genes in A genome (*G. arboreum*), D genome (*G. raimondii*), and A_t_D_t_ genomes (*G. hirsutum*). Sheet B: The comparative analysis of selection pressure (Ka/Ks) of *OMT* genes in *G. raimondii*, *G. arboreum*, and *G. hirsutum*.

**Additional file 8: Table S5.** Structural features of OMT genes.

## Data Availability

All data generated or analyzed during this study are included in this published article and its supplementary information files.

## Abbreviations

aa: amino acid
DPA: Days post anthesis
CCoAOMT: Caffeoyl coenzyme A 3-O-methyltransferase
CMTs: C-methyltransferases
COMTs: Caffeic acid 3-O-methyltransferases
NMTs: N-methyltransferases
OMT: O-methyltransferase
kDa: kilodalton
GO: Gene ontology
KEGG: Kyoto encyclopedia of genes and genomes
Ka: the number of non-synonymous substitutions per non-synonymous site
Ks: the number of synonymous substitutions per synonymous site
CSSLs: Chromosome segment substitution lines
RILs: Recombinant inbred lines
SAM: S-adenosyl-l-methionine
Ga: *Gossypium arboreum*
Gh: *Gossypium hirsutum*
Gr: *Gossypium raimondii*
qRT-PCR: quantitative real-time polymerase chain reaction
