# Supplementary figures and images for: Multi-responses of O-methyltransferase genes to salt stress and fiber development of Gossypium species
Source: BMC Plant Biol. 2021 Jan 11;21:37. doi: 10.1186/s12870-020-02786-6 (PMC7798291; doi:10.1186/s12870-020-02786-6)

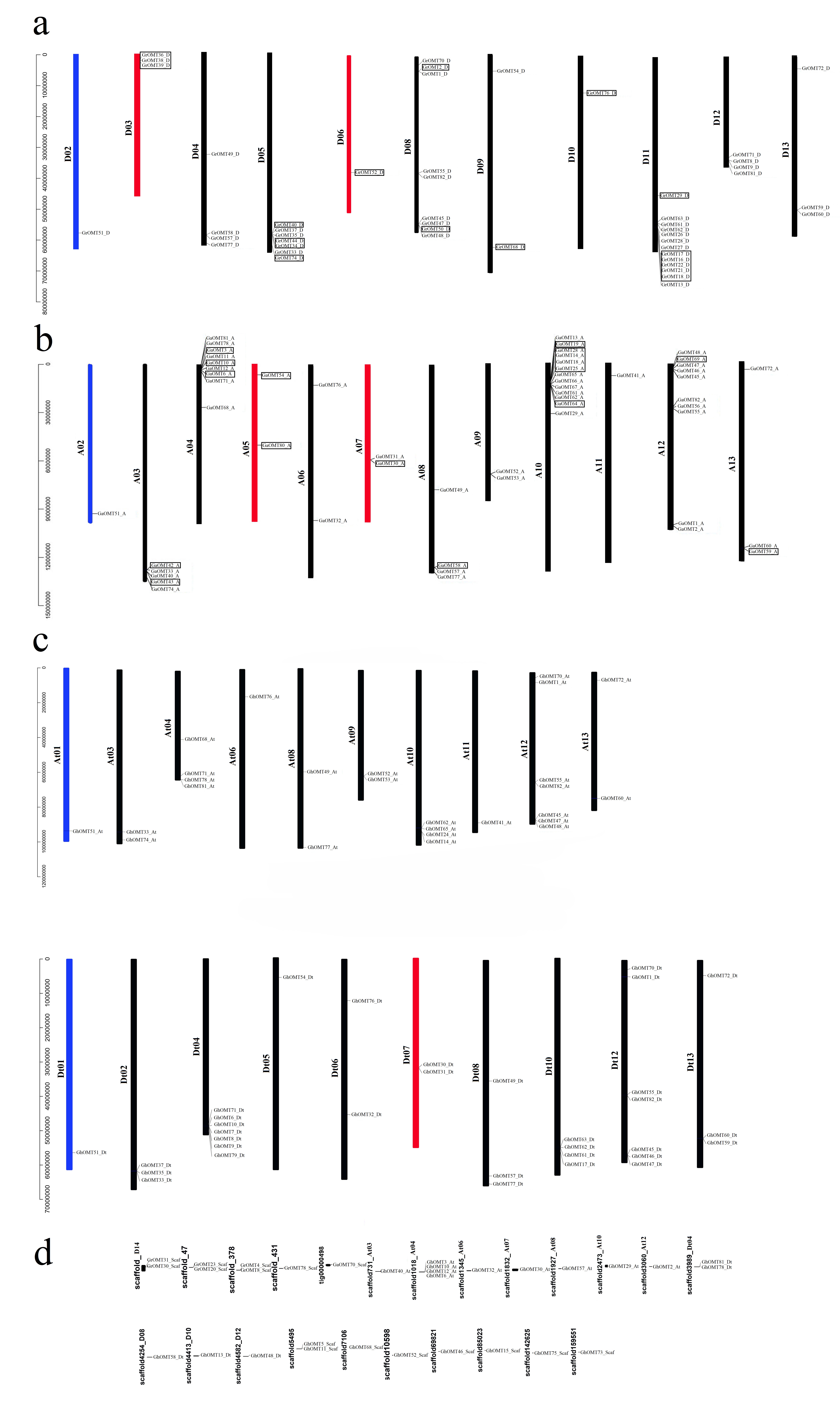

Supplement: Supplementary file 1 — Additional file 1: Figure S1. Chromosomal distribution of OMT genes in the genomes of Gossypium species. a: in D genome (G. raimondii), b: in A genome (G. arboreum), c:in AtDt genomes (G. hirsutum), d: in scaffolds of the three genomes. The genes in boxes in A and D genomes represent that their homologous genes in At and Dt sub-genomes are missing, while the genes in boxes in At and Dt sub-genomes represent that their homologous genes in A and D genomes are missing. [file 12870_2020_2786_MOESM1_ESM.jpg]

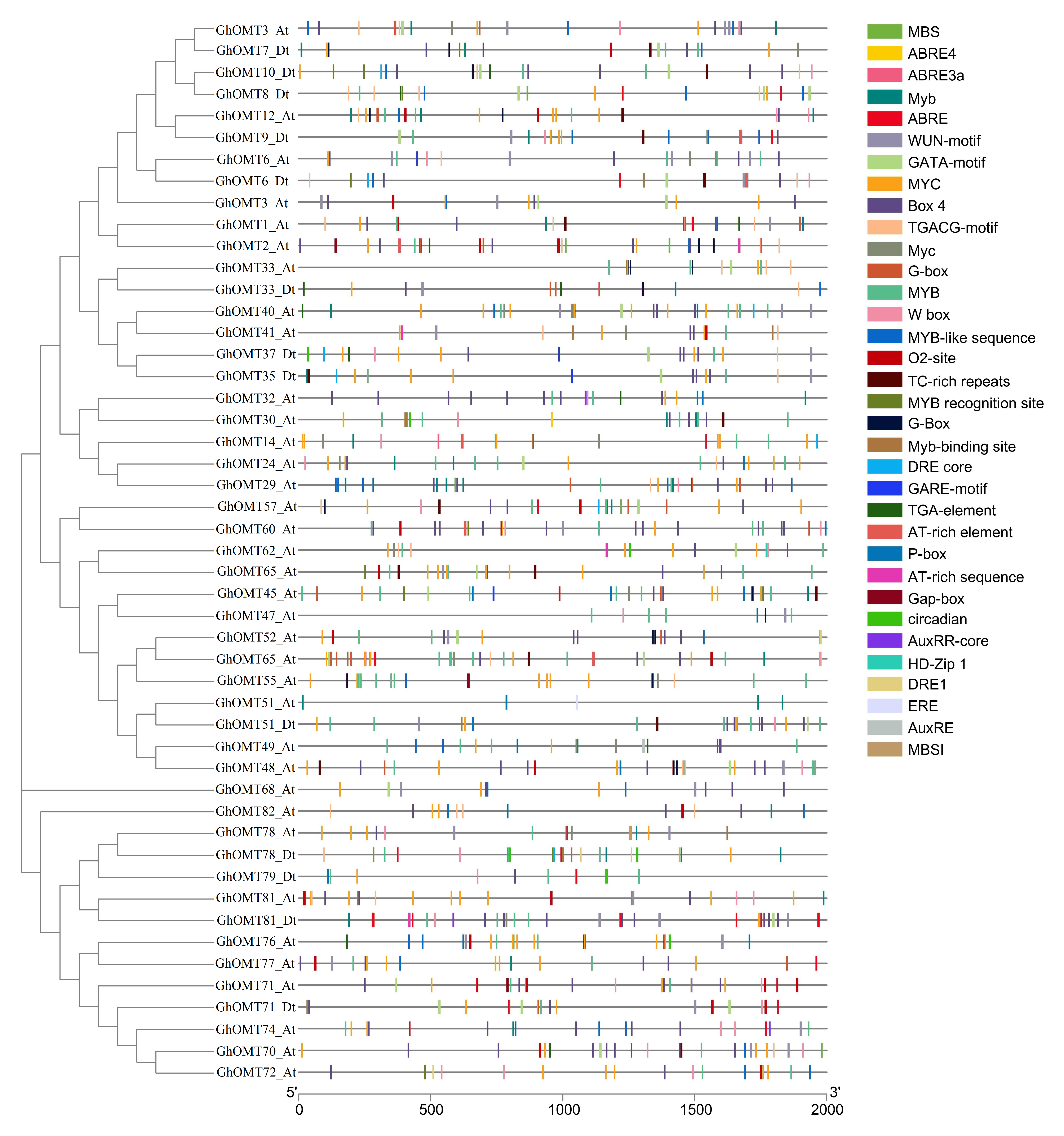

Supplement: Supplementary file 3 — Additional file 3: Figure S3. Identification of cis-regulatory elements of OMT genes. [file 12870_2020_2786_MOESM3_ESM.png]
