# Supplementary material for: Multi-responses of O-methyltransferase genes to salt stress and fiber development of Gossypium species
Source: BMC Plant Biol. 2021 Jan 11;21:37. doi: 10.1186/s12870-020-02786-6 (PMC7798291; doi:10.1186/s12870-020-02786-6)
Supplement: Supplementary file 4 — Additional file 4: Table S1. Detail Information of plant materials used for RNA-seq data acquisition. [file 12870_2020_2786_MOESM4_ESM.docx]

**Table S1. Detail Information of plant materials used for RNA-seq data acquisition**

| **population** |  | **Line** | **Material** | **characteristics** | **Species** | **Accessions** | **Institution** | **Reference** |
| --- | --- | --- | --- | --- | --- | --- | --- | --- |
|  |  | TM-1 | Standard line | Standard | *G. hirsutum* | PRJNA248163 | NAU | Zhang et al., 2015 |
| sGK9708 × 0-153 | RILs | 0-153 | Paternal line | Excellent fiber, low yield | *G. hirsutum* | PRJNA542946 | ICR, CAAS | Sun et al., 2012; Zhang et al., 2015, 2016, 2017; Jamshed et al., 2016 |
|  |  | sGK9708 | Maternal line | fair fiber quality, good yield | *G. hirsutum* |  |  |  |
|  |  | 69307 | A RIL | Higher fiber strength and length | *G. hirsutum* |  |  |  |
|  |  | 69362 | A RIL | Poor fiber quality and yield | *G. hirsutum* |  |  |  |
| CCRI45 × Hai1 | CSSLs | CCRI45 | Recurrent parent line | Higher yield, lower fiber quality | *G. hirsutum* | SRP084203 | ICR, CAAS | Shi et al.2015 |
|  |  | Hai1 | Donor parent line | *Verticillium* wilt resistant, excellent fiber | *G. barbadense* |  |  |  |
|  |  | MBI7561 | A CSSL | Higher fiber strength | CSSL |  |  |  |
|  |  | MBI7747 | A CSSL | Higher fiber strength | CSSL |  |  |  |
|  |  | MBI7285 | A CSSL | Lower fiber strength | CSSL |  |  |  |
| CCRI36 × Hai1 | CSSLs | CCRI36 | Recurrent parent line | Higher yield, lower fiber quality | *G. hirsutum* | SRX2843778 | ICR, CAAS | Shi et al.2015 |
|  |  | Hai1 | Donor parent line | *Verticillium* wilt resistant, excellent fiber | *G. barbadense* |  |  |  |
|  |  | MBI9915 | A CSSL | Higher fiber strength and length | CSSL |  |  |  |
|  |  | MBI9749 | A CSSL | Higher fiber strength and length | CSSL |  |  |  |
|  | | | | | *G. arboreum* | PRJNA179447 | ICR, CAAS | Du et al. 2018 |
|  | | | | | *G. raimondii* | PRJNA79005 | ICR, CAAS | Wang et al. 2012 |
