# Supplementary material for: Multi-responses of O-methyltransferase genes to salt stress and fiber development of Gossypium species
Source: BMC Plant Biol. 2021 Jan 11;21:37. doi: 10.1186/s12870-020-02786-6 (PMC7798291; doi:10.1186/s12870-020-02786-6)
Supplement: Supplementary file 5 — Additional file 5: Table S2. Sequences of Primer Pairs of five selected OMT family genes for qRT-PCR verification. [file 12870_2020_2786_MOESM5_ESM.docx]

**Table S2. Sequences of Primers Pairs of five selected OMTfamily genes in qRT-PCR verification**

| **Gene name** | **primer** | **Primer sequence** |
| --- | --- | --- |
| Gh_Histone3 | F | TCAAGACTGATTTGCGTTTCCA |
|  | R | GCGCAAAGGTTGGTGTCTTC |
| GhOMT49_At | F | TCGGATGAGGAAGCCAACTTA |
|  | R | GAAGGACTGAGGCACTGGTGA |
| GhOMT48_At | F | TTCTCTGCAATCTCTTCTTTT |
|  | R | AATATCTGGTGTGATGAAACT |
| GhOMT10_Dt | F | AATAGCTGTCGTTAAATGTGC |
|  | R | TGTCGGCAATCCCAAGCTCAA |
| GhOMT49_Dt | F | GCTACGAAGCTTTGCCGGAC |
|  | R | CCCACCAGGATTGTGAGCCA |
| GhOMT70_At | F | AACTACGAGCTGGGTCTGC |
|  | R | ATCCGTGGTTCTTTTCATCTTCCA |
